# Complete suppression of viral gene expression is associated with the onset and progression of lymphoid malignancy: observations in Bovine Leukemia Virus-infected sheep

**DOI:** 10.1186/1742-4690-4-51

**Published:** 2007-07-23

**Authors:** Makram Merimi, Pavel Klener, Maud Szynal, Yvette Cleuter, Claude Bagnis, Pierre Kerkhofs, Arsène Burny, Philippe Martiat, Anne Van den Broeke

**Affiliations:** 1 Laboratory of Experimental Hematology, Institut Jules Bordet, Université Libre de Bruxelles (ULB), 1000 Brussels, Belgium; 2 Institute of Pathological Physiology, Charles University, Prague, Czech Republic; 3 Etablissement Français du Sang, 13009 Marseille, France; 4 CERVA-CODA, 1180 Uccle, Belgium

## Abstract

**Background:**

During malignant progression, tumor cells need to acquire novel characteristics that lead to uncontrolled growth and reduced immunogenicity. In the Bovine Leukemia Virus-induced ovine leukemia model, silencing of viral gene expression has been proposed as a mechanism leading to immune evasion. However, whether proviral expression in tumors is completely suppressed *in vivo *was not conclusively demonstrated. Therefore, we studied viral expression in two selected experimentally-infected sheep, the virus or the disease of which had features that made it possible to distinguish tumor cells from their nontransformed counterparts.

**Results:**

In the first animal, we observed the emergence of a genetically modified provirus simultaneously with leukemia onset. We found a Tax-mutated (Tax_K303_) replication-deficient provirus in the malignant B-cell clone while functional provirus (Tax_E303_) had been consistently monitored over the 17-month aleukemic period. In the second case, both non-transformed and transformed BLV-infected cells were present at the same time, but at distinct sites. While there was potentially-active provirus in the non-leukemic blood B-cell population, as demonstrated by *ex-vivo *culture and injection into naïve sheep, virus expression was completely suppressed in the malignant B-cells isolated from the lymphoid tumors despite the absence of genetic alterations in the proviral genome. These observations suggest that silencing of viral genes, including the oncoprotein Tax, is associated with tumor onset.

**Conclusion:**

Our findings suggest that silencing is critical for tumor progression and identify two distinct mechanisms-genetic and epigenetic-involved in the complete suppression of virus and Tax expression. We demonstrate that, in contrast to systems that require sustained oncogene expression, the major viral transforming protein Tax can be turned-off without reversing the transformed phenotype. We propose that suppression of viral gene expression is a contributory factor in the impairment of immune surveillance and the uncontrolled proliferation of the BLV-infected tumor cell.

## Background

It is widely accepted that the majority of cancers if not all result from a combination of multiple cellular events leading to malignancy after a prolonged period of clinical latency. Alterations in the cell itself however may not be sufficient to drive full transformation and evidence has emerged that the immune system is playing a critical role in the control of cancer progression. Although the propensity of tumor cells to evade immune attack is well documented [1-3], there is little direct experimental evidence suggesting a correlation between immune evasion through virus- or oncogene-silencing and the onset of overt leukemia.

Sheep are particularly interesting as a large animal model for studying certain aspects of cancer biology. Compared to murine tumor models, information gained from large animal outbred populations such as sheep can be expected to be more informative about human malignancies [4]. Furthermore, sheep develop B-cell leukemia and lymphoma after experimental transmission of BLV, a virus belonging to the deltaretrovirus family, which encompasses HTLV-1 and -2 and STLVs [5-7]. Finally, in contrast to most rodent leukemia models in which a short mean latency precedes the aggressive acute phase, the ovine BLV-associated leukemia effectively recreates the temporal events that occur during the initiation and progression of chronic leukemia such as ATL and B-CLL in human.

In the model of BLV-induced leukemia and lymphoid tumors, viral infection and tumor progression can be monitored over time following injection with either naked proviral DNA or virus-producing cells [8,9]. BLV-infected sheep consistently develop tumors after a 6-month to 4-year period of latency. The pre-leukemic phase of infection includes the expansion of infected surface immunoglobulin M-positive (sIgM^+^) B-cells with proviral insertion at multiple sites, whereas a unique integration site represents the molecular signature of the malignant B-cell clone found in each individual after the onset of overt leukemia/lymphoma. Unlike simple retroviruses, which induce tumors by expressing viral products or by proviral insertional mutagenesis, complex oncoretroviruses such as HTLV-1 and BLV induce tumors using mechanisms which involve Tax, the viral transactivator. Tax deregulates signal transduction pathways, acts through the transcriptional modification of host genes and interactions with cellular proteins which create a cellular environment favoring aneuploidy and DNA damage [10-13]. Although Tax is an essential contributor to the oncogenic potential of both viruses, there is compelling evidence that expression of Tax is not sufficient for transformation. Furthermore, the presence of deletions and mutations in tumor-associated proviral sequences, including *tax*, suggests that neither virus nor Tax expression are required for the maintenance of the transformed phenotype [8,14,15].

BLV and HTLV-1 infection are both characterized by low or undetectable viral expression *in vivo *but cells isolated from an infected individual during the pre-malignant phase spontaneously express viral proteins *in vitro *[16,17]. However, in B-cell tumors isolated from BLV-infected sheep and cell lines that were derived from these tumors, we previously observed the presence of a silent provirus [8,15,18]. We raised the hypothesis that silencing of viral genes might be a strategy to circumvent effective immune attack. Because in BLV-infected sheep from earlier studies, the malignant cells were not easily distinguishable from their non-transformed infected counterparts, we studied viral expression in two selected BLV-infected individuals the virus or the disease of which had features that made it possible to separate tumor cells from non malignant cells. We found a correlation between the complete suppression of provirus expression and tumor onset, providing experimental evidence that virus and Tax silencing are critical if not mandatory for progression to overt malignancy.

## Results

### Sheep S2531: a case illustrating tumor-associated virus silencing by a genetic mechanism

**Figure 1:**
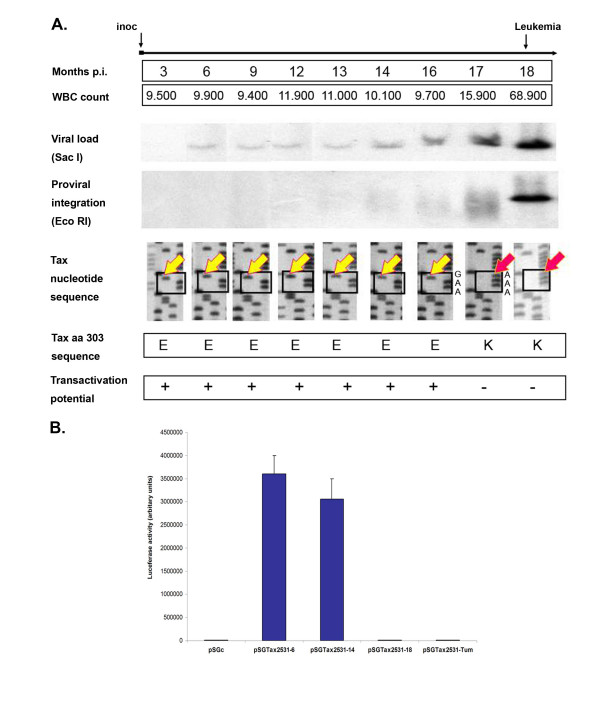
**Follow-up of sheep S2531: silencing occurssimultaneously with the onset of leukemia**. (A) Blood samples were collected from S2531 at regular time intervals over a 18-month period from the time of inoculation to the leukemic stage and examined for several parameters. WBC counts per mm^3 ^are indicated. Provirus load and integration were examined by Southern blot hybridization of *SacI*- and *EcoRI*-digests respectively, showing increasing provirus load and the progression from polyclonal to monoclonal integration as leukemia develops. The nucleotide sequence of the 3' end of the proviral *tax *DNA is illustrated by a polyacrylamide gel autoradiography of dideoxynucleotide sequenced PCR-amplified DNA. Boxes highlight nucleotides at positions 8149, 8150 and 8151 of the BLV sequence [29]. Arrows indicate the nucleotide identified at position 8149: a G at pre-leukemic stages (yellow arrow); a G to A transition at the time of the first documented WBC increase (17-month post-infection, red arrow). The resulting amino acid at position 303 of the corresponding Tax proteins is shown below. The transactivation potential of the putative S2531 proviral Tax proteins were examined in a luciferase reporter assay following co-transfection of HeLa cells with the pSGTax_2531 _expression vectors containing *tax *sequences cloned from S2531 PBMCs collected at different times post-infection and the reporter plasmid pLTR-Luc as detailed in B. "+" indicates a luciferase activity equivalent to that resulting from transfection with the wild-type pSGTax; "-" indicates the background level activity similar to that obtained when the empty expression vector pSG5 is co-transfected with pLTR-Luc. (B) Luciferase assay reflecting the transactivation potential of a selection of four S2531-derived *tax *sequences. Each pSGTax_2531 _construct containing the different S2531-derived *tax *sequences downstream of the CMV promoter was used in HeLa co-transfection with pLTR-Luc which expresses the firefly luciferase under the control of the BLV-LTR promoter. Luciferase activities were measured in cell lysates 42 h posttransfection and were normalized to protein concentrations as previously described [19]. Results are represented as histograms indicating basal luciferase activities (arbitrary units). pSGTax_2531–6 _and pSGTax_2531–14 _contain sequences amplified from PBMCs isolated during the aleukemic stage, 6 and 14 months post-inoculation respectively; pSGTax_2531–18 _contains *tax *sequences from leukemic PBMC isolated 18 months post-inoculation, and the pSGTax_2531-tum _construct resulted from the insertion of lymphoma-derived *tax *sequences collected 18 months post-infection. pSGc is the empty control vector. Values represent the means of the results of triplicate samples. The results from a representative experiment of four independent experiments are shown.

Sheep S2531 was injected with PBMCs isolated from S19, a sheep that had been inoculated in a previous study with YR2_LTaxSN_, a BLV-infected tumor B-cell line carrying both a silent Tax_K303_-mutated transactivation-deficient BLV provirus and a MoMuLV-derived retroviral vector expressing a functional Tax protein [8]. In S2531, antibodies to p24, the BLV capsid protein, were detected two weeks post-inoculation and persisted over time, suggesting that productive infection with a functional wild-type virus was taking place. Sequence analysis of the BLV provirus integrated in PBMCs isolated from S2531 demonstrated the presence of a replication-competent provirus characterized by a wild-type *tax *sequence (Fig. 1A), identical to that initially identified in the S19 PBMCs used in the inoculum. At position 303 of the Tax protein (309 aa), we identified a glutamic acid (E) resulting from a A_8149 _to G_8149 _transition which was shown to originate from homologous recombination between the transduced LTaxSN vector-derived wild-type *tax *(Tax_E303_) and the YR2-derived mutated *tax *sequence (Tax_K303_), consistent with our earlier studies of BLV-infected animals from the cohort to which S19 belongs [8]. In S2531, the Tax_E303 _replication-competent provirus was identified throughout the 17-month aleukemic period, characterized by normal WBC counts and a polyclonal integration pattern of the provirus, the hallmark of a non-transformed BLV-infected B-cell population (Fig. 1A, Proviral integration, *EcoRI*). S2531 developed a fatal B-cell leukemia as well as lymphoma eighteen months post-infection. This acute phase was characterized by the development of localized B-lymphoid tumors, as well as increasing WBC counts up to 68,900/mm^3^, a significantly increased virus load resulting from the proliferation of the malignant B-cell clone (Fig. 1A, Viral load *Sac I*) and a monoclonal integration pattern of the provirus in both the leukemic PBMCs and the lymphoid tumors. Sequence analysis revealed that, in contrast to the observations with PBMCs isolated at the aleukemic stage, the provirus identified in the malignant B-cell clone was a Tax_K303_-mutated replication-deficient provirus carrying an A at position 8149 (Fig. 1A, red arrows).

Expression vectors for Tax_2531 _were then constructed by exchanging the wild-type *tax *sequence in pSGTax with the PCR-amplified *tax *DNA from either pre-leukemic (position 8149 = G) or leukemic (position 8149 = A) S2531 samples respectively. HeLa cells were co-transfected with each pSGTax_2531 _construct together with the pLTRLuc reporter plasmid containing the firefly luciferase gene under the control of the BLV promoter as previously described [19]. Luciferase activities examined 42 hours post-transfection of pSGTax_2531 _constructs from samples 17-months post-inoculation were not significantly different from background levels generated by the control vector pSGc, confirming the transactivation-deficient phenotype associated with the genetic change observed in the tumor-derived proviral *tax*. As expected, constructs expressing *tax *sequences isolated from earlier samples, before the onset of leukemia, were consistently positive (Fig. 1A,B). Furthermore, two naïve sheep injected with the cloned S2531 proviral DNA isolated from leukemic cells failed to seroconvert and BLV-specific PCR was consistently negative, conclusively demonstrating that the tumor-associated S2531 provirus was non functional (data not shown). Thus, in S2531, while functional provirus had been consistently monitored over the 17-month aleukemic period, we exclusively found the transactivation-deficient provirus in both the peripheral lymphoid tumors and the blood isolated after progression to the acute leukemic phase. Finally, we examined whether the silent replication-deficient provirus might have been present as a minor form in the inoculum used to infect S2531. Therefore, we subcloned the PCR-amplified *tax *products obtained with DNA extracted from S19 PBMCs in the pCRScript^®^-SK(+) vector system (Stratagene) and sequenced multiple *tax *clones. Among a total of twenty sequenced clones we found two clones the sequence of which corresponded to the mutated *tax *(Tax_K303_), suggesting that besides wild-type replication-competent provirus (Tax_E303_) a minor population of replication-deficient provirus was present in the cells that served to infect S2531 (data not shown). Although it remains to be understood how and where a transactivation-deficient provirus was able to persist in S2531 before eventually giving rise to a transformed B-cell, our data show that while functional provirus was the major replicative form present over the pre-malignant stage, a transactivation-deficient provirus was selected after progression to acute leukemia. This *in vivo *follow-up strongly suggests that switching off Tax and virus expression is associated with the onset of full-blown malignancy.

### Sheep S267: a case illustrating tumor-associated virus silencing by an epigenetic mechanism

**Figure 2:**
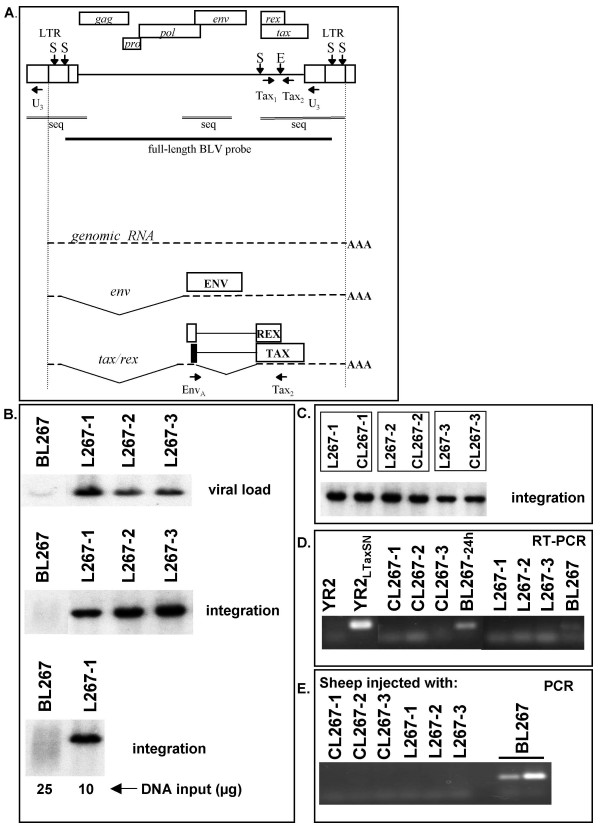
**Sheep S267: non-transformed blood-derived B-cells carry a potentially active provirus while virus and Tax expression are completely suppressed in the the co-existing malignant lymphoma B-cells**. (A) Diagram of the BLV L267 provirus and major transcripts. The two LTRs and the *gag*, *pro*, *pol*, *env*, *tax*, and *rex *genes are represented. Vertical arrows indicate restriction sites in the L267 provirus: S, *SacI*; E, *Eco*RI. The position and direction of the PCR primers are indicated on the provirus map. The horizontal bar indicates the 8.4 kb-long region that was used as probe. Double lines represent the sequenced regions. The genomic, *env*, and *tax/rex *transcripts are represented below. Alternatively spliced RNAs are not shown. The translation products of the singly- and doubly-spliced transcripts and the positions of the RT-PCR primers are indicated. (B) Southern blot analysis following hybridization with a full-length BLV probe of *SacI*-digested DNA isolated from blood (BL267) and lymphoma (L267-1, -2 and -3) cells collected from S267 twenty nine months post-infection. *SacI *is indicative of the proviral load (upper row). Southern blot analysis of *EcoRI*-digested DNA indicates the presence of a single monoclonally-integrated provirus for all three lymphoma (L267) whereas the blood-derived BL267 cells display a polyclonal integration pattern (middle and lower panels). *EcoRI*-cleaved DNA generates two virus-host junction fragments for each integrated L267 provirus as illustrated in the diagram. Shown here in each lane are the fragments containing the 5' flanking genomic region. (C) Southern blot analysis of *EcoRI*-digested DNA isolated from the lymphoma (L267-1, -2, -3) and the cell lines derived from each of these lymphoma (CL267-1, -2, -3) cultured for four weeks. (D) RT-PCR analysis of RNA isolated from lymphoma-derived cell lines (CL267), 24 h-cultured blood-derived lymphocytes (BL267-24 h), fresh lymphoma (L267) and freshly isolated blood-derived lymphocytes (BL267). EnvA/Tax2 primers for the detection of the doubly-spliced *tax/rex *RNA were used. In the controls YR2 and YR2_LTaxSN_, provirus is silent and active respectively. (E) PCR analysis using BLV *tax*-specific primer pair Tax1/Tax2 of DNA isolated from sheep inoculated with the various S267-isolated B-cell populations: six sheep were inoculated using either cultured (CL267) or fresh (L267) transformed B-cells, two sheep were injected with nontransformed PBMCs (BL267).

Although a proportion of the proviruses isolated from BLV-induced tumors carry genetic alterations including mutations and deletions, the vast majority of proviruses found in ovine tumors display a wild-type sequence. To determine whether silencing is unique to genetically-modified proviruses and thus rather an exception, or whether expression of structurally-intact proviruses found in tumor cells is also suppressed and thus the rule, we studied a second case, sheep S267, selected from an experimental cohort previously inoculated with cloned full-length wild-type proviral DNA [9]. While the majority of sheep from previous studies by others and our group developed both leukemia and lymphoma as a result of BLV infection, sheep S267 developed multiple peripheral lymphoid tumors (called lymphoma hereafter) in the absence of leukemia. Provirus was present in circulating B-cells, but WBC counts remained at a normal level (11,450 per mm^3 ^at the time of autopsy, 29 months post-infection). In sheep S267, it was thus possible to separate the infected non-transformed (blood) and infected transformed (lymphoma) B-cells. Each individual lymphoma (L267) consisted of an identical clonal population of transformed sIgM^+ ^B-cells carrying a single monoclonally-integrated BLV provirus, whereas the PBMCs (BL267) exhibited a non-transformed population characterized by random polyclonal provirus integration (Fig. 2A,B). The freshly-isolated lymphoma cells L267-1, -2, -3 and the B-cell cultures CL267-1, -2, -3 derived from these cells, displayed the same monoclonal integration pattern, suggesting that the cell lines were representative of the parental tumors (Fig. 2C). Whereas the lymphoma-derived CL267-1, -2, -3 cell lines were established from fresh L267-1, -2 and -3 cells in the absence of cytokines, culture of BL267 cells in similar conditions did not result in the outgrowth of transformed B-cells. Because cytokine-independent growth is a characteristic of B-cell transformation [12], our data strongly suggest that the blood-derived BLV-infected cells from S267 were not transformed.

B-cells freshly isolated from non-leukemic BLV-infected sheep spontaneously express viral proteins including Tax, whereas it is expected, if our hypothesis is correct, that tumor cells and the cell lines derived from these tumors harbor a silent provirus [8,15]. Using RT-PCR, we could not detect transcriptional activity in either the freshly isolated L267 lymphoma or the established CL267 transformed B-cell lines, whereas the blood-derived BL267 cells exhibited BLV-specific transcription (Fig. 2D). Importantly, the *in vivo *injection of naïve sheep with either fresh L267 lymphoma cells or lymphoma-derived CL267 cell lines did not result in productive infection, whereas injection of freshly-isolated BL267 cells, the blood-derived non-leukemic population, readily induced seroconversion to BLV-p24 as well as a detectable virus (Fig. 2E). Thus, while there is potentially-active provirus in the non-transformed blood-derived B-cells, provirus expression is silenced in the tumor B-cells as demonstrated by its incapacity to generate infection *in vivo*. Direct sequencing of selected regions of both the lymphoma- and blood-derived S267 proviruses including *tax*, the *pol/env *region required for *tax/rex *transcript expression as well as the complete 5'LTR (Fig. 2A) indicated identical sequences matching the injected wild-type proviral DNA [9,20-23]. Although it is possible that mutations in other regions might contribute to proviral extinction, our data suggest that tumor-associated silencing in S267 results from molecular mechanisms that are not linked to genetic changes. Interestingly, a sheep that had been infected with BL267 cells developed leukemia 25 month post-inoculation, characterized by 166,000 WBC/mm3 and a distinct provirus integration pattern as compared to that found in L267. Again, in the malignant clone of this animal, the BLV provirus was silent. A summary of these data is illustrated in Table 1. Overall, our observations in S267 reinforce the hypothesis that virus silencing plays a pivotal role in the establishment of a fully-transformed phenotype. Furthermore, these findings suggest that besides genetic changes, epigenetic mechanisms such as DNA methylation and chromatin modifications might be involved in tumor-associated virus latency.

**Table 1:** Characterization of PBMC- and lymphoma-derived B-cells isolated from sheep S267

**Cells isolated from:**	**Blood**	**Lymphoma**
provirus integration	polyclonal	monoclonal
cytokine-independent growth/capacity to derive cell lines	-	+
viral expression	+	-
provirus sequence	wild-type	wild-type
in vivo infectious potential	+	-

## Discussion

Using the BLV-associated ovine model of leukemia and based on the observations in two experimental sheep, we provide evidence for the role of virus and oncogene silencing as an important step in the onset of lymphoid malignancy. In the first animal, S2531, we identified a correlation between the genetic modification of the proviral structure and the emergence of leukemia. We found a Tax-mutated (Tax_K303_) replication-deficient provirus integrated into the genome of the malignant B-cell clone while recombinant functional provirus (Tax_E303_) had been consistently monitored over the aleukemic period. Although sequencing of individual *tax *clones identified the presence of a replication-deficient proviral form in the inoculum, our data provide no clues as to how this provirus might persist in the infected host. It will be important to sort out from our future studies whether the Tax_K303 _defective provirus found at the time of leukemia development in S2531 was already present in the pre-tumoral clone early after infection. A study is ongoing to answer this question, based on a BLV-specific inverse PCR technique for the detection of tumor-specific integration sites developed by Moules *et al*. [24]. Using this method, BLV-positive pre-malignant clones are detectable as early as two weeks after virus exposure. Whatever the mechanism responsible for this genetic modification, our observations suggest that switching off expression of Tax, the essential contributor to the oncogenic potential of BLV, is linked with the onset of acute leukemia. We propose that in this particular case, the mechanism by which the immune system destroys developing malignancies is evaded by the malignant cell by reducing its intrinsic immunogenicity, possibly through recombination-mediated virus silencing. In the second case, S267, both non-transformed and transformed BLV-infected cells were present at the same time, but at clearly distinct sites. While there was potentially-active provirus in the non-leukemic blood B-cell population, as demonstrated by *ex-vivo *culture and injection into naïve recipients, virus expression was completely suppressed in the malignant B-cells isolated from the lymphoid tumors despite the absence of genetic alterations in the proviral genome. This independent observation reinforces our previous conclusion and suggests that besides genetic alterations, epigenetic mechanisms might be involved in tumor-associated silencing. Altogether, our findings strongly support the hypothesis that switching-off viral gene expression, including Tax, the essential contributor to the oncogenic potential of BLV, is critical, if not mandatory, for progression to overt malignancy.

Sheep infected by BLV mount a strong immune response to viral antigens. Active killing of infected cells might play a decisive role in limiting BLV gene expression, but seems unable to prevent – or perhaps paradoxically favors – the development of a malignant clone harboring a silent provirus. It is tempting to assign our observations to the failure of the immune system to eliminate the infected cell given the absence of proper expression of immunogenic proteins, in this case Tax. Tax is the major target of CTLs in HTLV-associated disease [25], and we found significant levels of Tax-specific CTLs in BLV-infected sheep (Van den Broeke, unpublished results). The lack of immunogenicity of naturally occurring tumors is often understood in terms of a suboptimal condition in the tumor microenvironment to generate protective immunity, regulatory T-cell activity, dendritic cell dysfunction, production of suppressive factors such as IL-10, or changes in the pattern of antigen expression [1,3,26], but so far there was no example of complete suppression of tumor antigen expression, especially if this antigen is the major transforming protein.

The demonstration in S2531 of a link between the interruption of the long clinical latency and the complete suppression of viral expression suggests that silencing is a late event in the multi-step process leading to the uncontrolled growth of a transformed B-cell clone and the onset of the fatal acute stage of the disease. Early after infection, cells that do not express viral proteins might have a survival advantage because they escape CTLs, but such cells will not outgrow the cells that express virus because of the absence of functional Tax protein capable of transactivating the host cell pathways responsible for enhanced B-cell proliferation. However, if virus silencing occurs when the cell has undergone sufficient events to reach a point of no return, impairment of immune surveillance might allow the uncontrolled proliferation of this fully-transformed B-cell clone. Whatever the mechanism – genetic or epigenetic – it is critical for achieving complete silencing of all viral genes. Cellular changes that have occurred during the process of leukemogenesis are such that even the Tax oncoprotein can be turned off without reversing the transformed phenotype. Loss of Tax and virus expression has been extensively documented in HTLV-1-associated disease and both genetic and epigenetic silencing mechanisms have been described [13,27,28]. This study in sheep contributes to the further understanding of tumor-associated silencing. In particular, the analysis of sequential samples of the same individual from pre-tumoral to overt leukemia and the documentation of the timing of the Tax expression reduction are unique. Our findings are in strong contrast with observations in other viral-associated malignancies including HPV-, EBV-, and HBV-associated cancers, as well as tumors mediated by simple oncornaviruses that all require sustained oncogene or transforming gene expression. This observation also raises a major concern for the application of effective anti-tumor immunotherapy. CTLs to the oncogenic protein might be effective when elicited during the chronic pre-leukemic stage, but would be irrelevant for eliminating malignant cells that do not longer express the initially-immunogenic target antigen after tumor progression.

## Methods

### Animals and animal samples

All sheep were housed at the Centre de Recherches Vétérinaires et Agrochimiques (Brussels, Belgium). Experimental procedures were approved by the Comité d'Ethique Médicale de la Faculté de Médecine ULB and were conducted in accordance with national and institutional guidelines for animal care and use. S2531 was inoculated intradermally with 10^7 ^PBMCs isolated from a BLV-infected animal (S19) described earlier [8]. S267 was injected with naked proviral DNA of an infectious BLV variant (pBLVX3C) [9], isogenic to the full-length wild-type 344 provirus used for *in vivo *infection of sheep [9,20-23]. Blood was collected in EDTA-containing tubes and PBMCs were isolated using standard Ficoll-Hypaque separation. S267 lymphoid tumors were collected at necropsy, minced through a nylon mesh cell strainer (Becton-Dickinson) to obtain single-cell suspensions. Sheep used for injection with S267-derived cell populations were inoculated with 2 × 10^7 ^BL267, L267, or CL267 respectively. Anti-p24 antibody titers and viral load were determined as previously described [8].

### Cell cultures

PBMCs and single cell suspensions isolated from BLV-infected sheep were cultured at a concentration of 10^6 ^cells/ml in OPTIMEM medium (Invitrogen) supplemented with 10% FCS, 1 mM sodium pyruvate, 2 mM glutamine, non-essential amino acids and 100 μg/ml kanamycin as previously described [8].

### Southern blot, PCR, RT-PCR and sequence analysis

Genomic DNA was prepared and analyzed by Southern blot and PCR analysis as previously described [8]. The nylon-bound *Sac I or EcoRI*-digested genomic DNAs were hybridized with a ^32^P-labeled BLV full-length proviral DNA probe (Fig. 2A). Primers for PCR were as follow (nucleotide positions according to Sagata [29]: Tax1 [7321–7340]: 5'-GATGCCTGGTGCCCCCTCTG-3', Tax2 [7604–7623]: 5'-ACCGTCGCTAGAGGCCGAGG-3', U3 [8599–8618]:5'-GCCAGACGCCCTTGGAGCGC-3'. Tax1-Tax2 and Tax1-U3 were paired together for proviral DNA detection and sequencing respectively. For RT-PCR experiments, total RNA was extracted using the Tripure reagent according to the manufacturer's protocol (Roche). 1 μg of RNA was reverse transcribed and amplified using the Titan RT-PCR system according to the protocol supplied by the manufacturer (Roche). Primers EnvA [4766–4787]: 5'-TCCTGGCTACTAACCCCCCCGT-3', and Tax2 were used for the detection of the 2.1 kb doubly-spliced *tax/rex *mRNA as previously described [8], generating a fragment of 482 bp (Fig. 2A). For provirus sequencing, amplification of selected regions was performed using the *Pfu *proofreading DNA polymerase (Stratagene) and the purified products were sequenced using the Thermosequenase radiolabeled terminator cycle sequencing method (GE Healthcare Biosciences).

### Constructs and luciferase assays

DNA extracted from PBMCs isolated from S2531 at different times post-infection was amplified using primers Tax1/U3. *Eco RI*-restricted products were inserted into pSGTax [30] for exchange with the wild-type sequence. Each pSGTax_2531 _construct was used in HeLa co-transfection with pLTR-Luc, and luciferase activities were measured as described [19]. pSGTax contains the wild-type *tax *downstream of the CMV promoter; pLTR-Luc expresses the firefly luciferase under the control of the BLV-LTR promoter.

Proviral DNA from S2531 leukemic cells was cloned by insertion of *EcoRI*-restricted genomic DNA into the Lambda Dash^® ^II vector (Stratagene) according to the manufacturer and used to evaluate the infectious potential in sheep.

## Abbreviations

ATL: Adult T-cell Leukemia; B-CLL: B-cell Chronic Lymphocytic Leukemia; BLV: Bovine Leukemia Virus; EBV: Epstein-Bar Virus; HBV: Hepatitis-B Virus; HPV: Human Papilloma Virus; HTLV-1: Human T-lymphotropic Virus-1; MoMuLV: Moloney Murine Leukemia Virus; PBMCs: Peripheral Blood Mononuclear Cells; STLV: Simian T-lymphotropic Virus; WBC: White Blood Cell.

## Competing interests

The author(s) declare that they have no competing interests.

## Authors' contributions

MM and PK set up the experiments, carried out most of the experimental work, and participated to the writing of the manuscript, MS participated in the transfection and luciferase assays, YC performed the cloning and sequencing experiments, PK was responsible for the follow-up of the animals, CB participated in the experimental design and analysis of retroviral vector-associated recombination events, AB and PM helped with the interpretation of the results and corrected the manuscript, AVDB was the principal designer of the study, coordinated its realization and the writing of the manuscript. All authors read and approved the final manuscript.
